# Author Correction: Fetal whole heart blood flow imaging using 4D cine MRI

**DOI:** 10.1038/s41467-020-20353-3

**Published:** 2020-12-14

**Authors:** Thomas A. Roberts, Joshua F. P. van Amerom, Alena Uus, David F. A. Lloyd, Milou P. M. van Poppel, Anthony N. Price, Jacques-Donald Tournier, Chloe A. Mohanadass, Laurence H. Jackson, Shaihan J. Malik, Kuberan Pushparajah, Mary A. Rutherford, Reza Razavi, Maria Deprez, Joseph V. Hajnal

**Affiliations:** 1 School of Biomedical Engineering & Imaging Sciences, King’s College London, London, SE1 7EH UK; 2 Department of Congenital Heart Disease, Evelina Children’s Hospital, London, SE1 7EH UK; 3 Centre for the Developing Brain, King’s College London, London, SE1 7EH UK

**Keywords:** Heart development, Paediatric research, Biomedical engineering, Imaging techniques

Correction to: *Nature Communications* 10.1038/s41467-020-18790-1, published online 5 October 2020.

The original version of this Article contained an error in the spelling of the author Reza Razavi, which was incorrectly given as Reza Rezavi. This has now been corrected in both the PDF and HTML versions of the Article.

