# Supplementary material for: Fetal whole heart blood flow imaging using 4D cine MRI
Source: Nat Commun. 2020 Oct 5;11:4992. doi: 10.1038/s41467-020-18790-1 (PMC7536221; doi:10.1038/s41467-020-18790-1)
Supplement: Supplementary file 1 — Supplementary Information [file 41467_2020_18790_MOESM1_ESM.docx]

**Fetal whole heart blood flow imaging using 4D cine MRI**

T.A Roberts, J.F.P van Amerom, et al.

**Supplementary Information**

| **ID** | **GA** | **Fetal weight (kg)** | **Clinical status** | **No. Stacks** |
| --- | --- | --- | --- | --- |
| **01** | 32^+1^ | 1.93 | dilated aortic root | 5 |
| **02** | 30^+6^ | 1.65 | volunteer (normal heart) | 5 |
| **03** | 24^+2^ | 0.74 | volunteer (normal heart) | 5 |
| **04** | 29^+6^ | 1.32 | right aortic arch | 5 |
| **05** | 31^+0^ | 1.82 | ventricular diverticulum | 6 |
| **06** | 32^+3^ | 1.99 | right aortic arch | 6 |
| **09** | 28^+0^ | 1.01 | volunteer (normal heart) | 5 |

**Supplementary Table 1: Fetal study subjects.** Columns denote: fetal case number (ID, consistent with van Amerom et al.^33^); fetal gestational age in weeks^+days^ (GA); fetal weight estimated from segmentation of structural scans (kg); number of multi-planar dynamic MR stacks used for reconstruction of 4D flow cine volumes (No. Stacks).


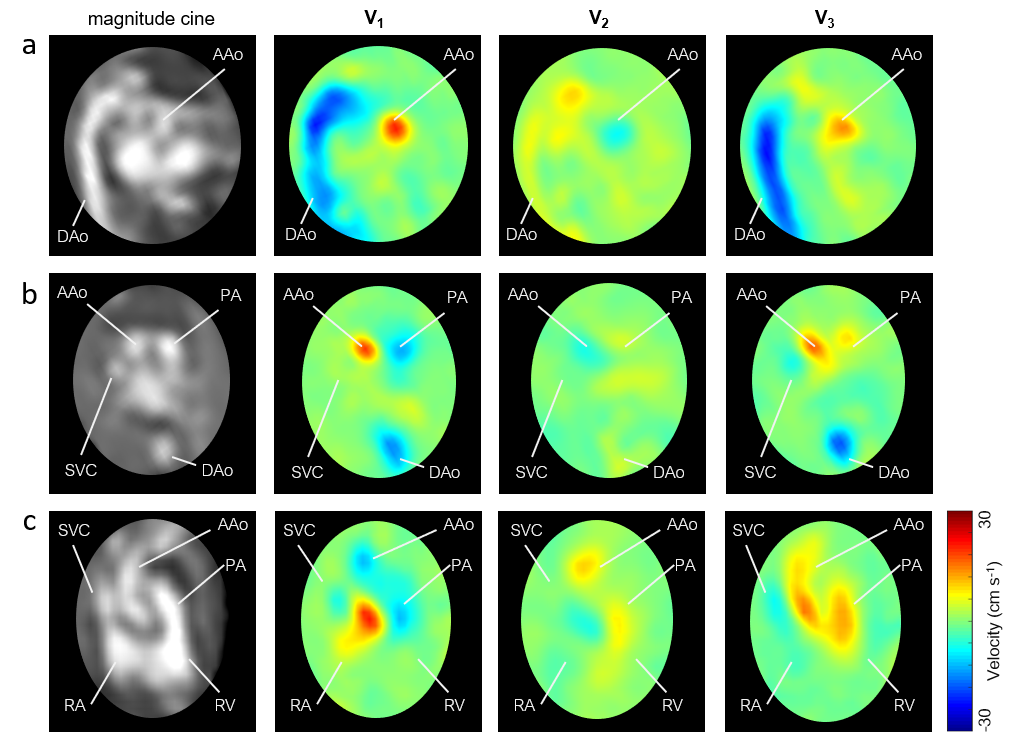


**Supplementary Figure 1: Cross-sectional views through reconstructed 4D velocity-component volumes.** Three different slices at systole taken from 4D magnitude volume and corresponding velocity-component maps in subject ID03 (healthy fetus, 24^+2^ weeks GA). Magnitude images (left column) are shown with corresponding maps showing the three orthogonal components of the velocity field (**V_1_**, **V_2_**, **V_3_**). Rows: (a) Aortic arch plane: increased and opposing velocity can be seen in the ascending aorta (AAo) and descending aorta (DAo). Anti-parallel velocity can be seen between the AAo and DAo in components **V_1_** and **V_3_**. (b) Three vessel view showing the AAo, DAo, pulmonary artery (PA) and superior vena cava (SVC). Increased velocity is seen in all vessels compared to background velocity. (c) Three chamber view of the right outflow tract showing the AAo, PA, SVC, right atrium (RA) and right ventricle (RV). Velocities with common directionality can be seen in component **V_3_**, while reduced, antiparallel velocities are seen in the SVC.
