# Supplementary material for: Fetal whole heart blood flow imaging using 4D cine MRI
Source: Nat Commun. 2020 Oct 5;11:4992. doi: 10.1038/s41467-020-18790-1 (PMC7536221; doi:10.1038/s41467-020-18790-1)
Supplement: Supplementary file 3 — Description of Additional Supplementary Files [file 41467_2020_18790_MOESM3_ESM.docx]

**Supplementary Movie Legends**

**Supplementary Movie 1:** Aortic arch view of a volumetric 4D flow cine render showing three-directional velocity vectors in subject ID03 (healthy fetus 24^+2^ weeks gestational age) overlaid on a 4D magnitude cine render. A still from this video with vessel labels overlaid can be seen in Figure 4a.

**Supplementary Movie 2:** Long-axis view of a volumetric 4D flow cine render showing three-directional velocity vectors in subject ID03 (healthy fetus 24^+2^ weeks gestational age) overlaid on a 4D magnitude cine render. A still from this video with vessel labels overlaid can be seen in Figure 4b.

**Supplementary Movie 3:** Right-sided of a volumetric 4D flow cine render showing three-directional velocity vectors in subject (healthy fetus 24^+2^ weeks gestational age) overlaid on a 4D magnitude cine render. A still from this video with vessel labels overlaid can be seen in Figure 4c. Note, velocity vectors in the aorta have been removed from this view for visual clarity of right-sided circulation.

**Supplementary Movie 4:** Aortic arch view of a volumetric 4D flow cine render showing three-directional velocity vectors in subject ID06 (right aortic arch fetus 32^+3^ weeks gestational age) overlaid on a 4D magnitude cine render. A still from this video with vessel labels overlaid can be seen in Figure 5a.

**Supplementary Movie 5:** Long-axis view of a volumetric 4D flow cine render showing three-directional velocity vectors in subject ID06 (right aortic arch fetus 32^+3^ weeks gestational age) overlaid on a 4D magnitude cine render. A still from this video with vessel labels overlaid can be seen in Figure 5b.
