# Supplementary figures and images for: Fetal whole heart blood flow imaging using 4D cine MRI
Source: Nat Commun. 2020 Oct 5;11:4992. doi: 10.1038/s41467-020-18790-1 (PMC7536221; doi:10.1038/s41467-020-18790-1)

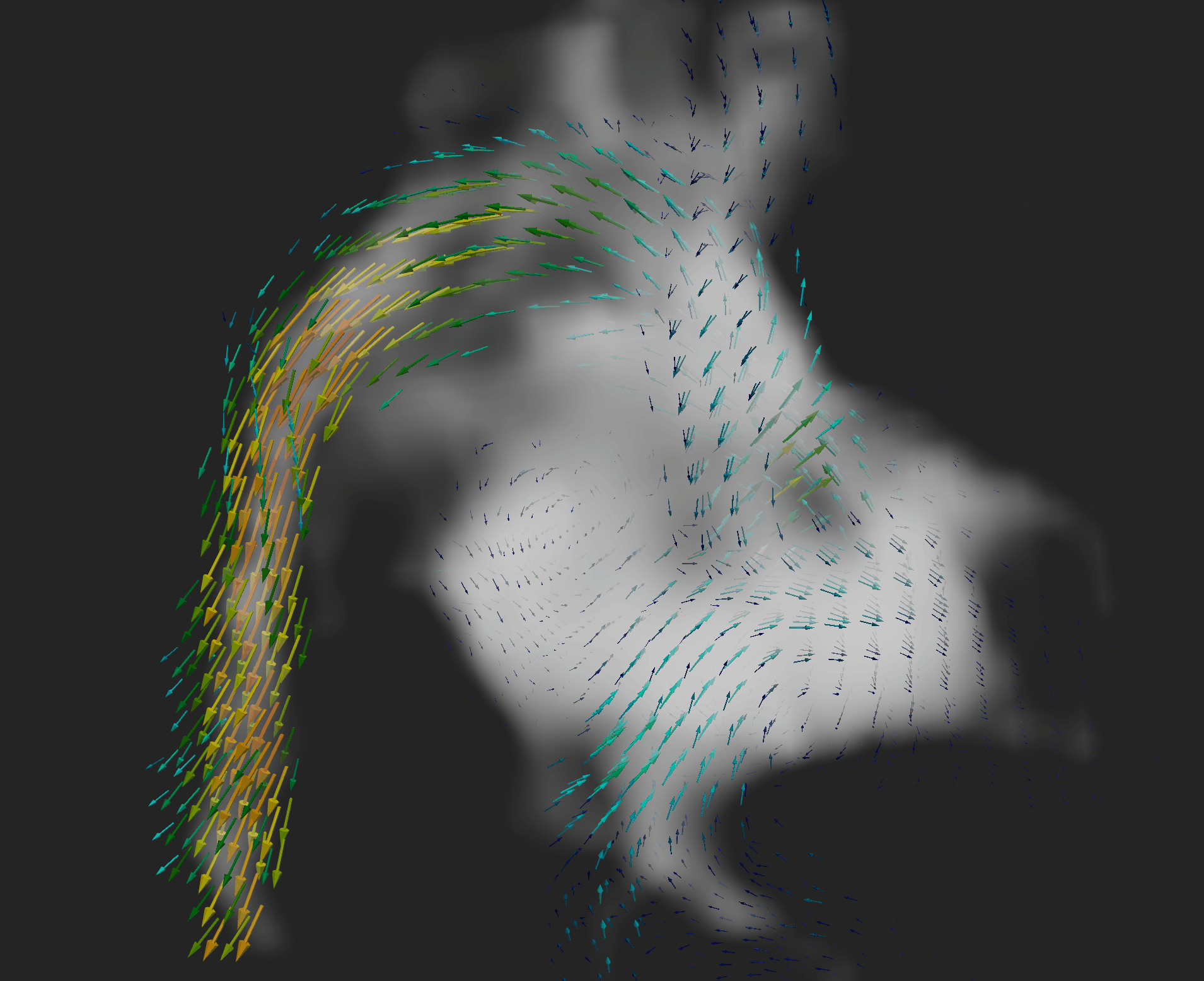

Supplement: Supplementary file 4 — Supplementary Movie 1 [file 41467_2020_18790_MOESM4_ESM.gif]

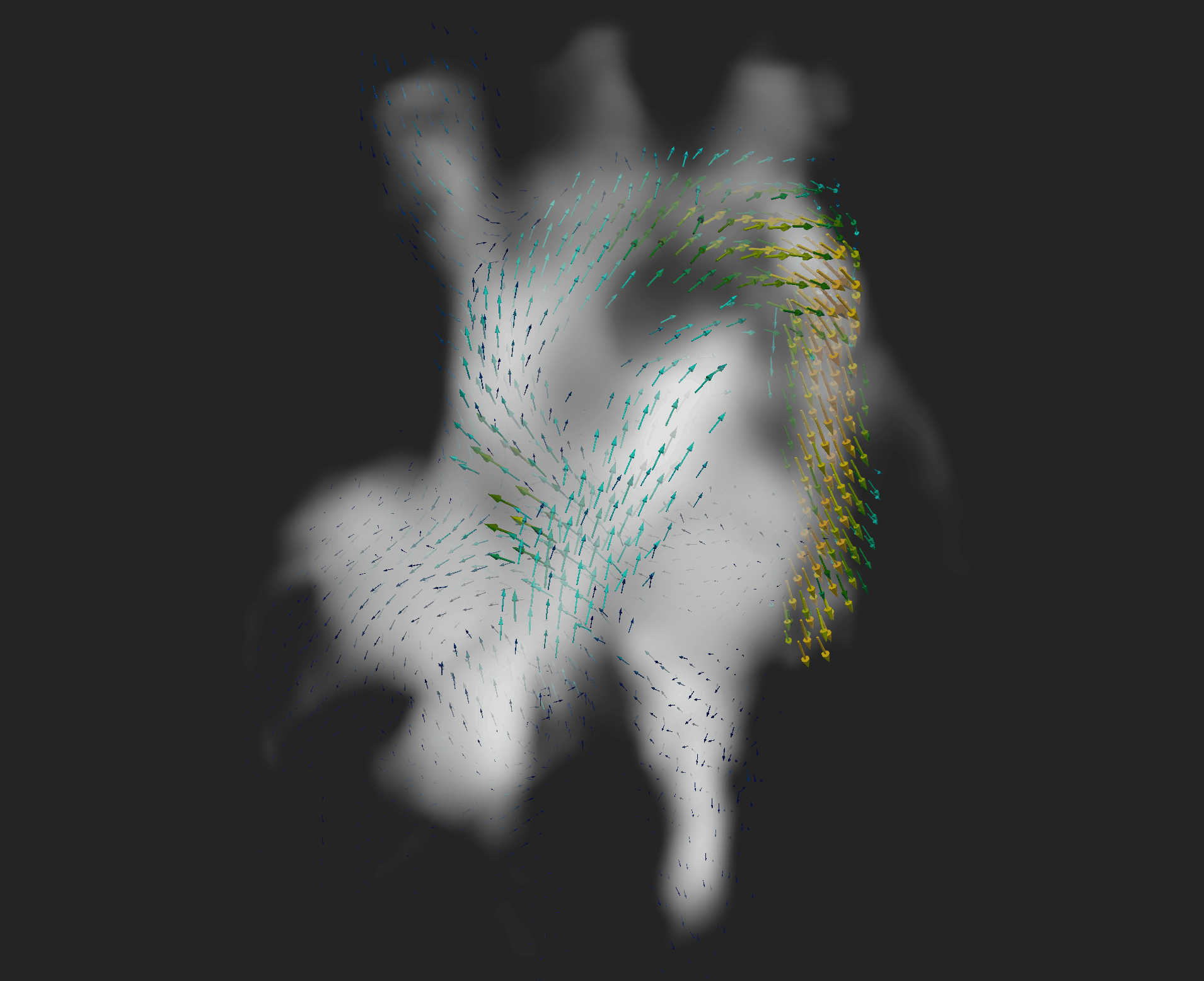

Supplement: Supplementary file 5 — Supplementary Movie 2 [file 41467_2020_18790_MOESM5_ESM.gif]

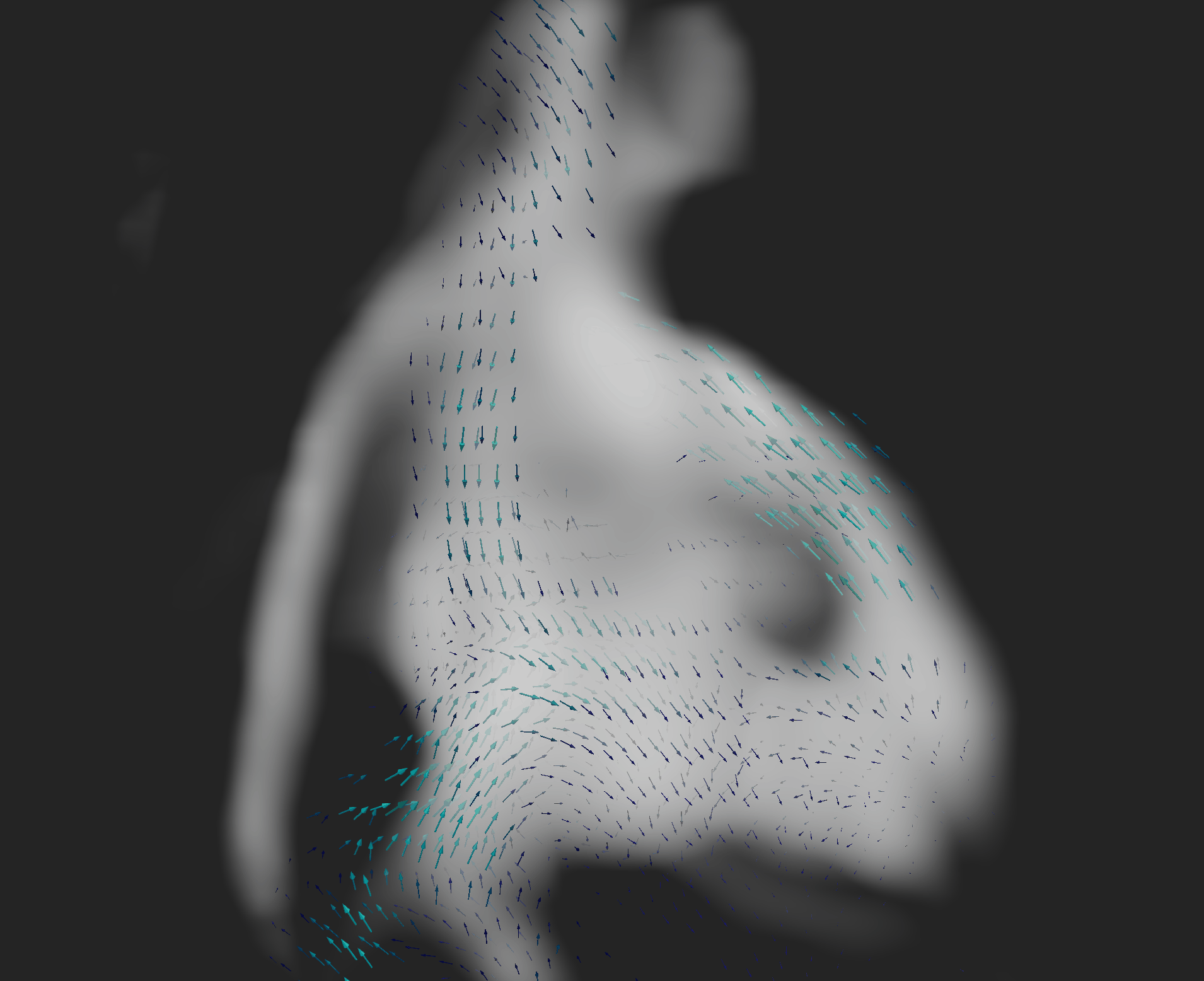

Supplement: Supplementary file 6 — Supplementary Movie 3 [file 41467_2020_18790_MOESM6_ESM.gif]

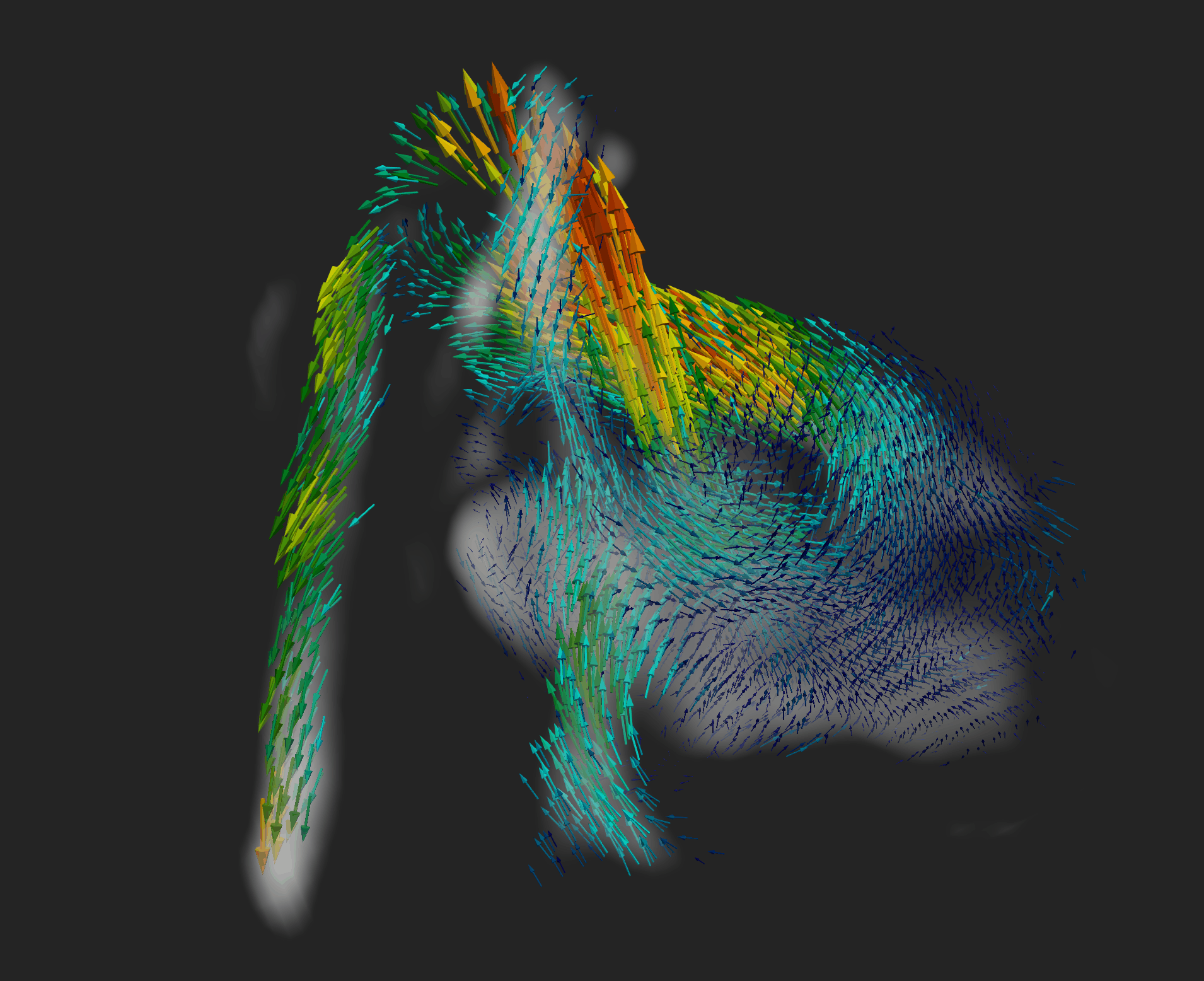

Supplement: Supplementary file 7 — Supplementary Movie 4 [file 41467_2020_18790_MOESM7_ESM.gif]

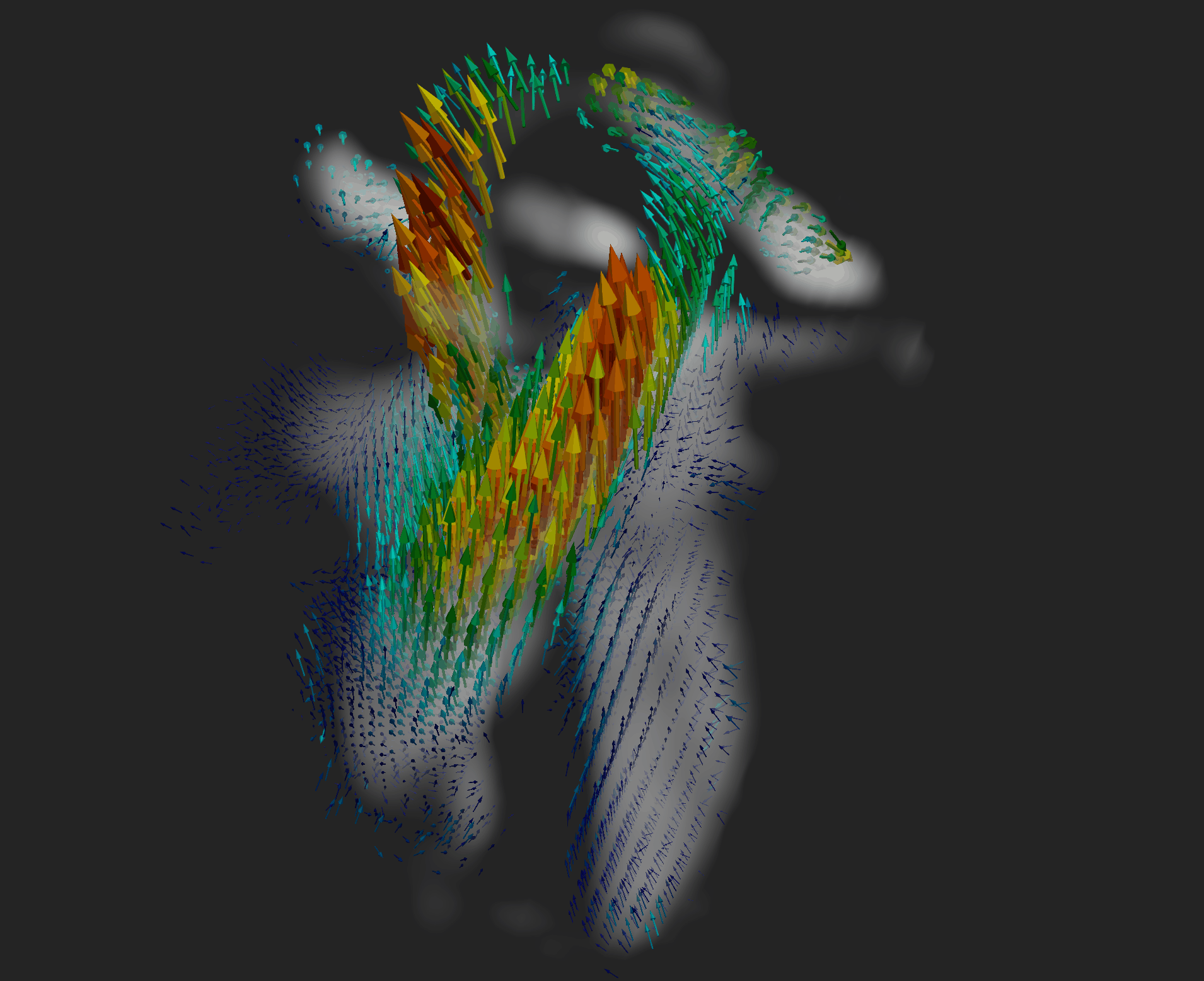

Supplement: Supplementary file 8 — Supplementary Movie 5 [file 41467_2020_18790_MOESM8_ESM.gif]
