# Supplementary material for: Fetal whole heart blood flow imaging using 4D cine MRI
Source: Nat Commun. 2020 Oct 5;11:4992. doi: 10.1038/s41467-020-18790-1 (PMC7536221; doi:10.1038/s41467-020-18790-1)
Supplement: Supplementary file 9 — Reporting Summary [file 41467_2020_18790_MOESM9_ESM.pdf]

nature research

Corresponding author(s): Thomas A Roberts
Last updated by author(s): Jul 29, 2020

Reporting Summary

Nature Research wishes to improve the reproducibility of the work that we publish. This form provides structure for consistency and transparency in reporting. For further information on Nature Research policies, see Authors & Referees and the Editorial Policy Checklist .

Statistics

For all statistical analyses, confirm that the following items are present in the figure legend, table legend, main text, or Methods section.

n/a	Confirmed	
☐	☒	The exact sample size (*n*) for each experimental group/condition, given as a discrete number and unit of measurement
☐	☒	A statement on whether measurements were taken from distinct samples or whether the same sample was measured repeatedly
☒	☐	The statistical test(s) used AND whether they are one- or two-sided<br>*Only common tests should be described solely by name; describe more complex techniques in the Methods section.*
☒	☐	A description of all covariates tested
☒	☐	A description of any assumptions or corrections, such as tests of normality and adjustment for multiple comparisons
☐	☒	A full description of the statistical parameters including central tendency (e.g. means) or other basic estimates (e.g. regression coefficient) AND variation (e.g. standard deviation) or associated estimates of uncertainty (e.g. confidence intervals)
☒	☐	For null hypothesis testing, the test statistic (e.g. *F*, *t*, *r*) with confidence intervals, effect sizes, degrees of freedom and *P* value noted<br>*Give P values as exact values whenever suitable.*
☒	☐	For Bayesian analysis, information on the choice of priors and Markov chain Monte Carlo settings
☒	☐	For hierarchical and complex designs, identification of the appropriate level for tests and full reporting of outcomes
☒	☐	Estimates of effect sizes (e.g. Cohen's *d*, Pearson's *r*), indicating how they were calculated

Our web collection on statistics for biologists contains articles on many of the points above.

Software and code

Policy information about availability of computer code

Data collection	Philips Ingenia MRI Scanner (Release 5 software)
Data analysis	ReconFrame MRecon-3.0.535 (GyroTools)<br>https://github.com/tomaroberts/synthflow_phantom (SHA1:3f4183c672c)<br>https://github.com/tomaroberts/fetal_cmr_4dflow (SHA1:38c18da4013c)<br>https://github.com/SVRTK/SVRTK/tree/CardiacVelocity4D (SHA1: 8182b9a7d98)<br>Note: the code is now collated in a single repository: https://github.com/mriphysics/fetal_cmr_4d<br><br>MATLAB 2018a // GraphPad Prism 6 // MITK Workbench (2016.11.0) // IRTK (v1.0) // MIRTK (v2.0.0) // MRTrix (v3.0_RC3-140-gd53818b7) // Paraview v5.4.1

For manuscripts utilizing custom algorithms or software that are central to the research but not yet described in published literature, software must be made available to editors/reviewers. We strongly encourage code deposition in a community repository (e.g. GitHub). See the Nature Research guidelines for submitting code & software for further information.

Data

Policy information about availability of data

All manuscripts must include a data availability statement. This statement should provide the following information, where applicable:

- Accession codes, unique identifiers, or web links for publicly available datasets
- A list of figures that have associated raw data
- A description of any restrictions on data availability

The data that support the findings of this study are available upon reasonable request from the corresponding author (T.A.R.). Patient data are not publicly available due to them containing information that could compromise research participant privacy or consent. An anonymised version of the data pertaining to subject ID03 is available from https://doi.org/10.6084/m9.figshare.c.4689437.

Field-specific reporting

Please select the one below that is the best fit for your research. If you are not sure, read the appropriate sections before making your selection.

☒ Life sciences ☐ Behavioural & social sciences ☐ Ecological, evolutionary & environmental sciences

For a reference copy of the document with all sections, see nature.com/documents/nr-reporting-summary-flat.pdf

Life sciences study design

All studies must disclose on these points even when the disclosure is negative.

Sample size	The patients in this study were recruited as part of the iFIND project (http://www.ifindproject.com/). The scanning protocol for the iFIND project encompasses many different scans which take up to 1 hour per patient. In a limited number of cases, we were able to acquire enough data (5 or more stacks of k-t SENSE MR images) to perform the 4D flow MRI framework in our paper. Under these constraints, this resulted in our sample size of n = 7.
Data exclusions	No data were excluded from the analyses.
Replication	The data were not replicated as patients were only scanned once as per the wider research protocol. This was a novel method development study. Validation of the method was performed in phantoms against gold-standard MRI and benchtop measurements. For in utero data, comparisons were made with existing literature measurements using other techniques; robust and comprehensive validation against Doppler ultrasound or quantitative 2D flow MRI techniques is extremely challenging.
Randomization	All subjects were included in a single group, including both normal fetuses and fetuses with congenital abnormalities. Based on prior experience, the abnormalities were not expected to greatly affect measured flow rates in the various vessels.
Blinding	For evaluation of the 4D Flow MRI data by expert readers, data was blinded prior to analysis. Blinding was not performed during data collection because the same imaging protocol was acquired regardless of clinical status of the fetus.

Reporting for specific materials, systems and methods

We require information from authors about some types of materials, experimental systems and methods used in many studies. Here, indicate whether each material, system or method listed is relevant to your study. If you are not sure if a list item applies to your research, read the appropriate section before selecting a response.

Materials & experimental systems

n/a	Involved in the study	
☒	☐	Antibodies
☒	☐	Eukaryotic cell lines
☒	☐	Palaeontology
☒	☐	Animals and other organisms
☐	☒	Human research participants
☒	☐	Clinical data

Methods

n/a	Involved in the study	
☒	☐	ChIP-seq
☒	☐	Flow cytometry
☒	☐	MRI-based neuroimaging

Human research participants

Policy information about studies involving human research participants

Population characteristics	All fetal subjects were aged between 24 to 33 weeks gestational age. Mothers were recruited as volunteers to the iFIND project (http://www.ifindproject.com/) at St Thomas' Hospital in London. Clinical assessment of fetuses (either diagnosed healthy or with congenital heart disease) was performed prior to imaging in this study. No population characteristic (e.g.: race, age, etc.) was biased for or against.
Recruitment	Participants were recruited as part of the Intelligent Fetal Imaging and Diagnosis (iFIND) project (http://www.ifindproject.com/). Potential biases may be related to recruiting patients within a large city-based, government funded hospital.
Ethics oversight	All imaging was performed with approval from the local NHS London Bridge Research Ethics Committee (REC 14/LO/1806) and all participants gave written informed consent prior to enrolment

Note that full information on the approval of the study protocol must also be provided in the manuscript.
